# A Comprehensive Review on Acanthamoeba Keratitis: An Overview of Epidemiology, Risk Factors, and Therapeutic Strategies

**DOI:** 10.7759/cureus.67803

**Published:** 2024-08-26

**Authors:** Diksha Garg, Sachin Daigavane

**Affiliations:** 1 Ophthalmology, Jawaharlal Nehru Medical College, Datta Meghe Institute of Higher Education and Research, Wardha, IND

**Keywords:** epidemiology and risk factors, therapeutic strategies, diagnostic methods, contact lens complications, corneal infection, acanthamoeba keratitis

## Abstract

*Acanthamoeba* keratitis (AK) is a rare but severe corneal infection caused by the free-living amoeba, *Acanthamoeba*, which is ubiquitously present in the environment. This condition predominantly affects contact lens wearers but can also occur in non-lens users, particularly those exposed to contaminated water or with compromised immune systems. AK is characterized by progressive corneal inflammation, epithelial defects, and ulceration, which can lead to significant visual impairment or blindness if not promptly diagnosed and treated. This review aims to provide a comprehensive overview of AK by synthesizing current knowledge on its epidemiology, risk factors, pathophysiology, clinical manifestations, diagnostic approaches, and therapeutic strategies. The review also highlights preventive measures and public health strategies to reduce the incidence of this debilitating condition. A detailed examination of existing literature was conducted, focusing on the global incidence of AK, demographic trends, and various risk factors such as contact lens use, environmental exposures, and immunity status. The review also delves into the pathophysiology of *Acanthamoeba* infection, the host immune response, and the challenges in distinguishing AK from other forms of infectious keratitis. Therapeutic strategies, including medical and surgical interventions, are analyzed, along with emerging treatments. The global incidence of AK has increased, particularly among contact lens users, due to poor hygiene practices and environmental exposures. Early diagnosis remains challenging, often leading to delayed treatment and poorer outcomes. Biguanides and diamidines are the mainstays of medical therapy, with surgical options considered in advanced cases. Emerging therapies, such as photodynamic therapy and antimicrobial peptides, show promise in enhancing treatment outcomes. AK poses a significant threat to ocular health due to its potential for severe visual impairment and the complexities associated with its diagnosis and treatment. Early recognition, appropriate management, and public health initiatives focused on prevention are crucial for improving patient outcomes. Ongoing research and a collaborative approach among healthcare providers are essential to advancing the understanding and management of AK.

## Introduction and background

*Acanthamoeba* keratitis (AK) is a rare but severe corneal infection caused by *Acanthamoeba*, a free-living amoeba ubiquitously found in the environment, including water, soil, and air [1]. This opportunistic pathogen can infect the cornea, leading to a painful and sight-threatening condition. AK primarily affects individuals who wear contact lenses, but it can also occur in non-lens wearers, particularly those exposed to contaminated water or those with compromised immune systems [2]. The disease is characterized by progressive corneal inflammation, epithelial defects, and ulceration, which can result in significant visual impairment or even blindness if not promptly diagnosed and treated [3].

The significance of this review lies in the increasing incidence of AK and the challenges associated with its diagnosis and management. AK often presents similarly to other types of infectious keratitis, making early and accurate diagnosis difficult. Misdiagnosis or delayed treatment can lead to severe complications and poor visual outcomes [4]. Given the potential for severe morbidity, it is crucial for ophthalmologists, optometrists, and other healthcare providers involved in eye care to have a thorough understanding of AK. This review aims to provide a comprehensive overview of AK, synthesizing current knowledge and recent advancements to aid in better diagnosis, treatment, and prevention of this debilitating condition [5].

The primary objectives of this review are to summarize the current knowledge on the epidemiology of AK, including global incidence rates and demographic trends. It aims to elucidate the various risk factors associated with AK, such as contact lens wear, environmental exposures, and immunity status. Additionally, this review explores the pathophysiology of *Acanthamoeba* infection and the host immune response. It discusses clinical manifestations, diagnostic approaches, and challenges distinguishing AK from other forms of infectious keratitis. The review also analyzes established and emerging therapeutic strategies for managing AK, including medical and surgical interventions. Furthermore, it evaluates long-term outcomes and factors influencing prognosis and highlights preventive measures and public health strategies to reduce AK incidence.

## Review

Epidemiology

*Acanthamoeba* keratitis (AK) represents a significant public health concern, especially among contact lens wearers [1]. The global annual incidence of AK is estimated at approximately 23,561 cases, corresponding to a prevalence rate of about 2.9 cases per million individuals [6]. This accounts for roughly 2% of all corneal infections worldwide. Historically, the incidence was much lower, with estimates ranging from one to two cases per million contact lens users in the late 20th century. The increase in reported cases is attributed to greater awareness, improved diagnostic techniques, and the rising popularity of contact lenses [1]. There are notable regional variations in the incidence of AK. In North America, particularly in the United States, outbreaks have been linked to specific contact lens solutions, leading to heightened awareness and more frequent reporting of cases [7]. In Europe, studies have documented a rising trend in AK cases, with some regions experiencing a significant increase in incidence over the past few decades. For example, a nationwide survey in the UK showed a marked rise in cases from 1994 to 2018 [8]. Similarly, in Asia, incidence rates vary based on local practices concerning contact lens use and hygiene. Overall, the trend suggests a global increase in AK cases, likely due to the growing popularity of contact lenses and changes in lens care practices [9]. Demographic characteristics of affected populations indicate that AK predominantly impacts contact lens users. Most cases occur among individuals who do not follow proper hygiene practices, including daily and extended wear users [10]. While AK can affect individuals of any age, it is most commonly reported in young adults, particularly those aged 20 to 40 years. Geographic distribution also plays a role, with higher incidences reported in urban areas where contact lens use is more prevalent. Additionally, although AK primarily affects otherwise healthy individuals, those with compromised immune systems or pre-existing ocular conditions are at increased risk [10].

Pathophysiology

*Acanthamoeba* exhibits a biphasic life cycle consisting of two primary stages: trophozoites and cysts. Trophozoites are the active feeding stage that divides mitotically under favorable conditions, such as abundant food, neutral pH, and an optimal temperature of around 30°C [11]. Measuring 14-40 μm in diameter, trophozoites are responsible for causing infections. Cysts, in contrast, represent a dormant, environmentally resistant stage that forms in response to adverse conditions such as food scarcity, extreme pH or temperature, high cell density, or exposure to chemicals. Cysts have a double-layered wall and are 12-16 μm in diameter [12]. *Acanthamoeba* can infect humans through several routes, including the eye, respiratory tract, and skin. In contact lens wearers or individuals with corneal trauma, trophozoites may enter the eye and cause severe keratitis. Trophozoites or cysts can also be inhaled through the nasal passages and reach the lower respiratory tract, while broken skin or ulcers offer an entry point for the amoeba to invade the body. Upon entering the host, *Acanthamoeba* can cause various clinical syndromes depending on the infection route and the individual's immune status [13]. The most common manifestation of *Acanthamoeba* infection is keratitis, predominantly affecting contact lens wearers or those with corneal trauma. Without prompt treatment, this can lead to vision loss. Granulomatous amebic encephalitis (GAE) is a rare but fatal central nervous system infection, usually occurring in immunocompromised individuals. GAE is characterized by severe central nervous system dysfunction and rapid degeneration. Additionally, *Acanthamoeba* can disseminate hematogenously to cause skin lesions, sinusitis, pneumonia, and other systemic infections, primarily in immunocompromised hosts [14]. The host immune response to *Acanthamoeba* infection remains poorly understood. While serum antibodies against *Acanthamoeba* are common in healthy individuals, their role in protective immunity is uncertain. Cell-mediated immunity, particularly involving T cells, is likely critical in controlling the infection. Immunocompromised individuals - such as those with AIDS, organ transplants, cancer, or those on long-term steroid therapy - are at greater risk of developing severe, disseminated disease [15].

Risk factors

**Figure 1:**
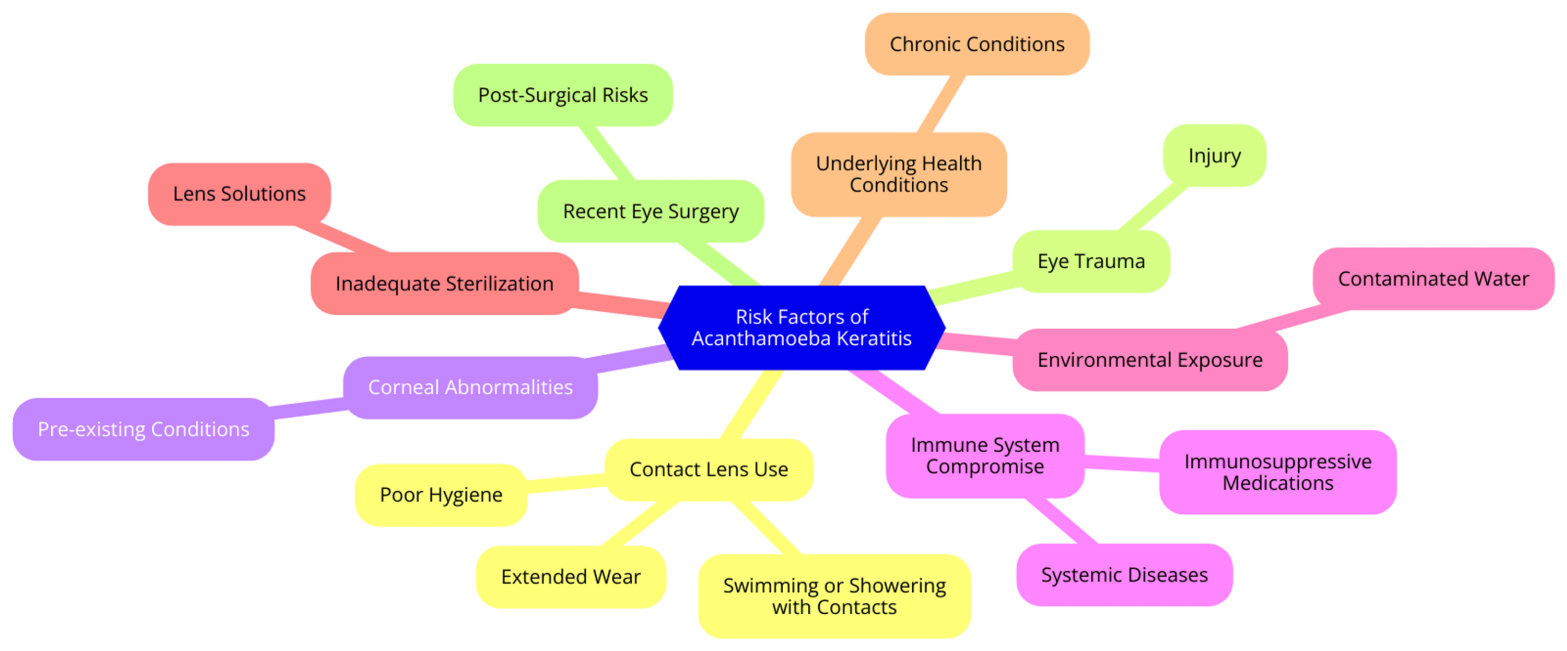
The risk factors for Acanthamoeba keratitis Image credit: Dr Diksha Garg

AK is a severe corneal infection that poses a significant risk, particularly to contact lens users. Understanding the risk factors associated with this condition is crucial for effective prevention and management [16]. One of the primary risk factors for AK is contact lens use, with approximately 90% of cases occurring in individuals who wear contact lenses. Poor hygiene practices notably increase the risk of infection. For example, rinsing lenses with tap water, using expired or inappropriate cleaning solutions, and failing to replace lenses as recommended can lead to contamination [17]. Extended-wear lenses, designed for continuous use, especially overnight, can compromise corneal health by reducing oxygen supply and creating a favorable environment for *Acanthamoeba* growth. The type of contact lenses used also plays a role; certain materials may present different risks, making it essential for users to follow care instructions specific to their lens type [2]. Environmental exposures are another significant factor in the development of AK. *Acanthamoeba* is commonly found in various water sources, including tap water, swimming pools, and hot tubs. Using tap water to rinse or store lenses can introduce the amoeba directly into the eye. Swimming pools and hot tubs, particularly poorly maintained ones, can harbor the organism, increasing infection risk [18]. Additionally, exposure to soil and dust, common in outdoor activities like gardening or sports, can pose a threat, as these environments contain *Acanthamoeba*. Any pre-existing corneal abrasions can further heighten the risk of infection [18]. Trauma to the eye and pre-existing ocular surface diseases are additional risk factors for AK. Corneal abrasions from foreign objects, scratches, or improper lens handling create entry points for *Acanthamoeba*. Individuals with ocular surface diseases, such as dry eye syndrome or other inflammatory conditions, may have compromised corneal integrity, making them more susceptible to infections [1]. Lastly, immunocompromised states significantly elevate the risk of developing AK. Individuals with systemic diseases such as diabetes, HIV/AIDS, or autoimmune disorders may have a weakened immune response, impairing their ability to combat infections. Those undergoing immunosuppressive therapies, including corticosteroids or chemotherapy, face an even greater risk, as these treatments further compromise the immune system's ability to defend against pathogens like *Acanthamoeba* [19]. The risk factors for AK are illustrated in Figure 1.

Diagnostic methods

Diagnosing AK requires a comprehensive approach integrating clinical examination, microbiological tests, molecular techniques, and imaging modalities [18-20]. Each method is vital for confirming the presence of *Acanthamoeba* and evaluating the extent of the infection [20-23]. The primary diagnostic tool for AK is the slit lamp examination. This technique allows visualization of corneal opacities, infiltrates, and the characteristic ring infiltrate associated with AK and helps assess the severity of the infection and overall corneal health [24]. Microbiological tests, including culture techniques and staining methods, are crucial for confirming the organism's presence. Corneal scraping followed by culture on non-nutrient agar with an *Escherichia coli* overlay can promote *Acanthamoeba* growth, though results may take several days. Staining methods, such as the Giemsa stain, can visualize *Acanthamoeba* cysts and trophozoites in corneal scrapings, offering rapid identification, though they are less definitive than culture [25]. Molecular techniques, particularly polymerase chain reaction (PCR) testing, provide highly sensitive and specific methods for detecting *Acanthamoeba* DNA in corneal samples. PCR offers rapid results and is especially useful when culture results are negative but clinical suspicion remains high. Next-generation sequencing (NGS) allows for detailed genetic analysis of corneal samples, identifying *Acanthamoeba* species and genotypes and providing insights into epidemiology and potential treatment responses [26]. In vivo confocal microscopy (IVCM) is a non-invasive imaging technique that offers real-time visualization of corneal layers, enabling the detection of *Acanthamoeba* cysts and trophozoites. IVCM is particularly valuable for early diagnosis and monitoring of treatment response [20]. The diagnosis of AK involves a combination of clinical evaluation and various diagnostic techniques. Each method contributes to a thorough understanding of the infection, guiding effective therapeutic strategies and improving patient outcomes. Early and accurate diagnosis is essential for effectively managing and preserving vision in affected individuals [20].

Therapeutic strategies

AK demands a comprehensive approach to treatment, integrating medical management, surgical interventions, and innovative therapies. Each component plays a vital role in addressing the complexities of this potentially sight-threatening infection [27]. Medical management of AK primarily involves antimicrobial agents. Biguanides, such as polyhexamethylene biguanide (PHMB) and chlorhexidine, are typically the first-line treatments due to their broad-spectrum antimicrobial properties and effectiveness against *Acanthamoeba*. Additionally, diamidines, including propamidine and hexamidine, are used for their anti-*Acanthamoeba* activity and can be combined with biguanides to enhance treatment efficacy [28]. Adjunctive therapies are also critical in managing AK. Corticosteroids may be cautiously prescribed to reduce inflammation and alleviate pain, though their use requires careful monitoring to prevent exacerbation of the infection. Pain management is essential, as patients often experience significant discomfort. Topical anesthetics and oral analgesics can help alleviate this pain, thus improving the overall quality of life for those affected [29]. In cases where medical management is insufficient, surgical interventions may be necessary. Debridement of the infected corneal epithelium can remove necrotic tissue and promote healing, particularly in severe cases of epithelial involvement. For patients with advanced disease or those who do not respond to medical therapy, penetrating keratoplasty (corneal transplant) might be required to restore vision. Alternatives such as lamellar keratoplasty or anterior lamellar keratoplasty may be considered based on the extent of corneal damage and individual patient circumstances [30]. Ongoing research explores novel and emerging therapies to enhance the treatment of AK. Photodynamic therapy, which uses light-activated compounds to target and eliminate *Acanthamoeba* cysts, shows promise as an adjunctive treatment that could improve outcomes. Additionally, antimicrobial peptides - naturally occurring molecules with antimicrobial properties - are under investigation for their potential efficacy against *Acanthamoeba*. Combination therapies involving multiple antimicrobial agents are also being studied to improve treatment efficacy and minimize the risk of resistance [28].

Prognosis and outcomes

Several key factors significantly influence the prognosis for AK. One of the most critical determinants is the diagnosis timing; patients receiving treatment within 14 days of symptom onset generally experience better visual outcomes [31]. Conversely, the use of corticosteroids before a definitive diagnosis can lead to poorer prognoses. This practice often results in delayed diagnosis, associated with decreased final vision and an increased need for therapeutic keratoplasty. Additionally, secondary complications such as cataracts and ocular hypertension can further complicate the clinical picture and negatively impact long-term outcomes [32]. Long-term outcomes for AK patients can vary widely. Studies indicate that approximately 39% of the affected eyes experience poor visual outcomes, including significant visual impairment or blindness. For those who require therapeutic keratoplasty due to severe corneal damage, the prognosis is generally less favorable [33]. Research shows that patients who manage AK successfully through medical therapy tend to achieve better visual acuity than those who undergo surgical interventions. For example, the final visual acuity for patients treated medically can be significantly better than those requiring surgical procedures, underscoring the importance of early and effective management [34]. Recurrence rates of AK have not been extensively studied, but the disease is known to be a challenging and persistent infection. Factors contributing to the risk of recurrence include incomplete treatment, drug resistance, and the presence of cysts that can evade standard therapies [31]. While specific recurrence rates are not well-quantified in the literature, the potential for reinfection highlights the need for careful monitoring and long-term follow-up for patients who have experienced AK. This vigilance is crucial for managing potential complications and ensuring optimal visual outcomes over time [1].

Prevention strategies

Preventing AK is essential, especially for contact lens users and individuals at higher risk. A comprehensive strategy encompassing public health initiatives, education on contact lens hygiene, guidelines for water exposure, and targeted recommendations for high-risk individuals can significantly reduce the incidence of this sight-threatening infection [1]. Public health initiatives are crucial for raising awareness about AK. Awareness campaigns should educate the public about symptoms, risk factors, and prevention strategies associated with AK. These campaigns can leverage various platforms, including informational brochures, social media, and community workshops. Establishing surveillance programs can also help monitor AK incidence and detect outbreaks, enabling timely public health interventions. Collaboration with eye care professionals, such as optometrists and ophthalmologists, ensures that healthcare providers are well-equipped to inform patients about the risks of AK and its prevention [35]. Education on proper contact lens hygiene is vital for minimizing the risk of AK among lens users. Users should be educated on following proper lens care protocols, including washing hands before handling lenses, cleaning and storing lenses in suitable solutions, and adhering to replacement schedules. Emphasizing the risks of sleeping in contact lenses and encouraging adherence to prescribed wear schedules is also important. Regular eye examinations should be promoted to monitor eye health and detect early signs of infection, allowing for prompt intervention if necessary [36]. Guidelines for water exposure are particularly important for contact lens wearers. Individuals should be advised to avoid wearing lenses while swimming, showering, or using hot tubs, as these activities increase the risk of *Acanthamoeba* exposure. If water exposure is unavoidable, using goggles can protect the eyes, and users should remove contact lenses before engaging in water-related activities. Additionally, recommending daily disposable lenses can further reduce contamination risks, as these lenses are discarded after a single use [37]. High-risk individuals, such as immunocompromised or working in environments with potential *Acanthamoeba* exposure, need tailored recommendations. For individuals with compromised immune systems - such as those with diabetes, HIV, or undergoing chemotherapy - specific advice should be provided to minimize infection risk. Similarly, outdoor workers in landscaping or construction should receive guidelines on eye protection and hygiene practices. Clear protocols should also be developed for individuals experiencing AK symptoms, ensuring they know when and how to seek medical attention promptly [15].

Future directions and research

AK is a rare but potentially sight-threatening corneal infection caused by the *Acanthamoeba* parasite. It primarily affects contact lens wearers and can also occur in non-contact lens users due to corneal trauma. AK is caused by the *Acanthamoeba* genus, a globally distributed unicellular protozoan parasite [1]. The epidemiology of AK indicates that it is an uncommon condition, with an estimated 1,500 cases annually in the United States. The two most frequently implicated species are *Acanthamoeba castellanii* and *Acanthamoeba polyphaga*, with over 23 genotypes identified, among which the T4 genotype is the most prevalent worldwide [38]. The risk factors for AK include contact lens use - especially when accompanied by improper lens hygiene and overnight wear - swimming or showering while wearing contact lenses, corneal trauma in non-contact lens users, and immunocompromised states. Clinically, AK presents with symptoms such as pain, redness, photophobia, and blurred vision, which may fluctuate in severity [2]. The diagnosis of AK typically involves a combination of slit lamp examination, IVCM, corneal scraping and culture, PCR testing, anterior segment optical coherence tomography (AS-OCT), and impression cytology. Treatment options range from conservative approaches with biguanides (e.g., polyhexamethylene biguanide), aromatic diamidines, and steroids to more invasive methods such as corneal crosslinking and, in severe cases, keratoplasty [39]. Prevention is crucial, particularly for contact lens users. Adhering to proper lens hygiene, avoiding water exposure, and using approved disinfection systems are essential measures to reduce the risk of AK [40]. Future research is vital for advancing the diagnosis, treatment, and understanding of AK. Current knowledge gaps include *Acanthamoeba*'s pathogenesis, the strains' genetic diversity, and the effects of co-infections on disease severity and treatment outcomes. Research directions should focus on diagnostic innovations, therapeutic strategies, and stem cell research for corneal repair [40]. Potential advancements in diagnostics and treatment may involve advanced imaging techniques, machine learning applications, and personalized medicine approaches. Addressing these areas is crucial for overcoming the challenges posed by AK and improving patient outcomes through enhanced diagnostic and therapeutic strategies [40].

## Conclusions

*Acanthamoeba* keratitis (AK) remains a significant ocular health challenge due to its potential for severe visual impairment and the complexities associated with its diagnosis and treatment. This review has comprehensively covered the epidemiology, risk factors, pathophysiology, clinical presentation, diagnostic methods, and therapeutic strategies of AK. It underscores the importance of early recognition and prompt, appropriate treatment to prevent serious complications and improve patient outcomes. Moreover, understanding the role of contact lens hygiene, environmental exposures, and immune status is crucial in mitigating the risk of AK. As advancements in diagnostic techniques and therapeutic options continue to evolve, there is hope for more effective management of AK. Public health initiatives and educational programs focused on prevention can also play a vital role in reducing the incidence of this sight-threatening condition. Ultimately, ongoing research and a collaborative approach among healthcare providers are essential to address the gaps in knowledge and enhance the care of patients affected by AK.
